# Influence of Enamel Matrix Derivative on Cells at Different Maturation Stages of Differentiation

**DOI:** 10.1371/journal.pone.0071008

**Published:** 2013-08-12

**Authors:** Richard J. Miron, Oana M. Caluseru, Vincent Guillemette, Yufeng Zhang, Anja C. Gemperli, Fatiha Chandad, Anton Sculean

**Affiliations:** 1 Faculté de medecine dentaire, Pavillon de médecine dentaire, rue de la Terrasse, Université Laval, Québec, Canada; 2 Department of Periodontology, School of Dental Medicine, University of Bern, Bern, Switzerland; 3 The State Key Laboratory Breeding Base of Basic Science of Stomatology (Hubei-MOST) & Key Laboratory of Oral Biomedicine Ministry of Education, School & Hospital of Stomatology, Wuhan University, Wuhan, People’s Republic of China; 4 Institut Straumann AG, Basel, Switzerland; INSERM U1059/LBTO, Université Jean Monnet, France

## Abstract

Enamel matrix derivative (EMD), a porcine extract harvested from developing porcine teeth, has been shown to promote formation of new cementum, periodontal ligament and alveolar bone. Despite its widespread use, an incredibly large variability among in vitro studies has been observed. The aim of the present study was to determine the influence of EMD on cells at different maturation stages of osteoblast differentiation by testing 6 cell types to determine if cell phenotype plays a role in cell behaviour following treatment with EMD. Six cell types including MC3T3-E1 pre-osteoblasts, rat calvarial osteoblasts, human periodontal ligament (PDL) cells, ROS cells, MG63 cells and human alveolar osteoblasts were cultured in the presence or absence of EMD and proliferation rates were quantified by an MTS assay. Gene expression of collagen1(*COL1*), alkaline phosphate(*ALP*) and osteocalcin(*OC*) were investigated by real-time PCR. While EMD significantly increased cell proliferation of all cell types, its effect on osteoblast differentiation was more variable. EMD significantly up-regulated gene expression of *COL1*, *ALP* and *OC* in cells early in their differentiation process when compared to osteoblasts at later stages of maturation. Furthermore, the effect of cell passaging of primary human PDL cells (passage 2 to 15) was tested in response to treatment with EMD. EMD significantly increased cell proliferation and differentiation of cells at passages 2–5 however had completely lost their ability to respond to EMD by passages 10+. The results from the present study suggest that cell stimulation with EMD has a more pronounced effect on cells earlier in their differentiation process and may partially explain why treatment with EMD primarily favors regeneration of periodontal defects (where the periodontal ligament contains a higher number of undifferentiated progenitor cells) over regeneration of pure alveolar bone defects containing no periodontal ligament and a more limited number of osteoprogenitor cells.

## Introduction

The goal of regenerative periodontal therapy is the reconstitution of the lost periodontal structures (i.e. the new formation of root cementum, periodontal ligament and alveolar bone) [1]–[3]. Results from preclinical and clinical research in the last decade have provided evidence for the biologic rationale and clinical applications of an enamel matrix derivative in periodontal wound healing/regeneration [4]. However, in light of the known functions of enamel matrix proteins (EMPs) during enamel formation (amelogenesis) [5], [6], a function in periodontal regeneration may seem controversial. In this context, it is important to know that EMPs, besides having roles in regulating the initiation and growth of hydroxyapatite crystals during the formation of enamel, are involved in the cell differentiation processes of many cell types [7]–[14]. Of particular interest are observations suggesting that specific amelogenin splice products may function as potential epithelial-mesenchymal signaling molecules during tooth development [15]–[18].

Initial in vitro studies demonstrated that PDL cells grown on dentin slices were unable to form cementum without specific EMPs demonstrating the critical importance of EMPs during cementogenesis [19]. These proteins have since been formulated into an enamel matrix derivative (EMD) for clinical application (Emdogain, Institut Straumann AG, Basel, Switzerland). The major components of EMD are amelogenins a family of hydrophobic proteins derived from different splice variants and controlled by post-secretory processing from a single gene that account for more than 95% of the total protein content [20]. These proteins self-assemble into supramolecular aggregates that form an insoluble extracellular matrix that function to control the ultrastructural organization of the developing enamel crystallites [20]. Other proteins found in the enamel matrix include enamelin, ameloblastin (also called amelin or sheathlin), amelotin, apin, and various proteinases [5], [6]. Although the role of EMPs in cell differentiation has been extensively investigated, large variability of effectiveness exists amongst in vitro and clinical studies. While a large number of studies have demonstrated that EMD promotes both cell growth (proliferation) and differentiation [21]–[34], others have failed to show any additional benefit towards differentiation [35]–[38] and a small number have demonstrated an inhibitory effect on either cell proliferation or differentiation [39]–[41].

One important factor that has not been investigated is the influence of cell type on osteoblast behaviour in response to treatment with EMD. Since a large number of different primary and cell-line derived osteoblasts have been utilised for in vitro studies (MC3T3E-1, rat calvarial osteoblasts, ROS osteoblasts, MG63 osteoblast cell line, primary human osteobasts and PDL cells), it is plausible that some of the observed variability is caused by the differentiation stage at which osteoblast were tested. Since EMPs are embryologically used to differentiate cells *early* in their differentiation process, it is logical to hypothesize that EMD would also have a more pronounced effect on cells early in their differentiated process and that culture conditions may generate variable in vitro results based on this phenomenon.

Therefore, the aims of the present study were 1) to test the effects of EMD on 6 different osteoblast cell types (both primary and cell-lines) at different stages in osteoblast differentiation; 2) To test the effect of cell passaging on the responsiveness of EMD by comparing the effects of EMD on primary human PDL cells having been passaged between 2 and 15 times; 3) To test the effects of EMD on MC3T3-E1 pre-osteoblasts that have gradually been differentiated down the osteoblast phenotype.

## Methods

### Coating with EMD

EMD was prepared according to Institut Straumann AG standard operating protocols. 30 mg of EMD was dissolved in 3 ml of 4°C sterile 0.1% acetic acid. For experiments, stock EMD was diluted 100X in 0.1 M carbonate buffer at 4°C giving a working solution of 100 µg/ml. 1 ml of EMD solution was poured onto 100 mg of tissue culture plastic in 24 well culture dishes and incubated overnight at 4°C. Following incubation, samples were rinsed with 1 ml phosphate buffered saline (PBS, pH 7.4, Catalogue #, 10010–023, Gibco) twice at 4°C.

### Osteoblast and PDL Cell Isolation

Primary human osteoblasts were obtained from an explant model as previously described [42]. PDL cells were obtained from the middle third portion of teeth extracted for orthodontic reasons as previously described [42]. RCOs were harvested by collagenase digestion as previously described [33]. Briefly RCOs were obtained from newborn rat calvariae following decapitation. Frontal, parietal and occipital bones were dissected and rinsed in α-MEM (Gibco, Grand Island, NY, USA). Minced tissue was digested twice for 15 min each in a mixture of collagenase/trypsin (3∶1; both purchased from Sigma-Aldrich) and the second digestion plated in tissue culture flasks using α-MEM supplemented with antibiotics (100 g/ml penicillin G, Sigma-Aldrich; 50 g/ml gentamicin, Sigma-Aldrich; 3 mg/ml amphotericin B, Gibco, Grand Island, NY, USA and 15% fetal bovine serum (Cansera, Rexdale, Ont., CAN). Osteoblasts were removed from the tissue culture plastic using a trypsin solution [0.25% trypsin (Gibco), 0.1% glucose, citrate-saline buffer (pH 7.8)]. MC3T3-E1 pre-osteoblasts (Sigma-Aldrich, Canada), ROS-17/2.8 cells and MG63 osteoblast-like cells were purchased as cell lines from ATCC, Rockville, MD, USA. All cells were grown in α-MEM medium supplemented with antibiotics (100 g/ml penicillin G; 50 g/ml gentamicin; 3 mg/ml amphotericin B, Gibco) and 15% fetal bovine serum (Cansera). Cells were removed from the tissue culture plastic using a trypsin solution [0.25% trypsin (Gibco), 0.1% glucose, citrate-saline buffer (pH 7.8)]. The maturation of each cell type was confirmed via real-time PCR as described in figure S1. Cells were seeded at a density of 10,000 cells per 24 well culture plate (Falcon) for cell proliferation experiments and 50,000 cells per well for real-time PCR and alizarin red experiments cultured in α-MEM medium supplemented with antibiotics and 15% FBS.

### Proliferation Assay

Cell proliferation was quantified using the CellTiter 96 One Solution Cell Assay (MTS) (Promega, Madison, WI, USA) as previously described [43]. Cells were seeded in 24-well plates at a density of 10,000 cells per well in 24 well culture plate. After 3 and 5 days, cells were washed with PBS and then incubated with 80 µL of CellTiter96 aqueous solution dissolved in 400 µL of PBS. After 4 h of incubation, cell viability was determined by measuring the absorbance at 490 nm on a 96-well plate-reader. Experiments were performed in triplicate with three independent experiments for each condition. Data (+/− SE) were normalized to control cells not seeded with EMD at 1 day for each cell type. Data were analyzed for statistical significance using 2-way analysis of variance (ANOVA) with Bonferroni test (*, p<0.05).

### Real Time RT-PCR

Total RNA was isolated from cells using TRIZOL reagent and RNAeasy Mini kit (QIAGEN) at time point 14 days for osteoblast differentiation analysis. Primer and probe sequences for genes encoding collagen 1α1 (*COL1*, Hs01028970_m1), alkaline phosphatase (*ALP*, Hs01029144_m1), osteocalcin (*OC*, also known as bone gamma-carboxyglutamic acid-containing protein (*BGLAP*), Hs01587814_g1) and Glyceraldehyde 3-phosphate dehydrogenase (*GAPDH*, Hs03929097_g1) were purchased as pre-designed gene expression assays (Applied Biosystems). Real-time RT-PCR was performed using 20 µl final reaction volume of TaqMan®’s One step Master Mix kit. RNA quantification was performed using a Nanodrop 2000c and 100 ng of total RNA was used per sample well. All samples were assayed in triplicate and 3 independent experiments were performed. The ΔΔCt method was used to calculate gene expression levels normalized to *GAPDH* values and normalized to control samples at 1 day. Data were analyzed for statistical significance using 2-way analysis of variance (ANOVA) with Bonferroni test (*, p<0.05).

### Alizarin Red Quantification

Alizarin red staining was performed to determine the presence of extracellular matrix mineralization after 21 days of cell culture. Cells were seeded at a density of 50,000 cells per well in 24 well culture plates onto control and EMD-coated tissue culture plastic. After 21 days, cells were fixed in 96% ethanol for 15 minutes and stained with 0.2% alizarin red solution in water (pH 6.4) at room temperature for 1 hour. Alizarin red was dissolved using a solution of 20% methanol and 10% acetic acid in water for 15 minutes. Liquid was then transferred to cuvettes and optical density was read on a spectrophotometer at a wavelength of 450 nm. Three independent experiments were performed each with 3 replicates for each condition. Data was analyzed for statistical significance using one-way analysis of variance with Tukey’s test (*, p<0.05).

### PDL Cell Passaging Experiment

PDL cells were grown to confluency and then passaged using trypsin solution (Invitrogen). At cell passage 2, 5, 10 and 15 cells were trypsinized and seeded at a density of 10,000 cells per well in 24 well culture plates (Falcon) for cell proliferation experiments and 50,000 cells per well for real-time PCR experiments with or without EMD as previously described. Three independent experiments were performed each with 3 replicates for each condition. Data were analyzed for statistical significance using 2-way analysis of variance (ANOVA) with Bonferroni test (*, p<0.05).

### MC3T3-E1 Cell Differentiation Experiment

The mice pre-osteoblast cell line MC3T3-E1 was used to determine the influence of cell differentiation down the osteoblast lineage. Due to the ability for this cell-line to undergo spontaneous osteoblast differentiation induced by cell-cell contacts once confluency is reached under standard in vitro conditions [44], [45]. These cells were left in T-75 flasks for 0, 7, 14 and 28 days to determine the effect EMD might have following their gradual differentiation. At desired time points, cells were trypsinized and seeded at a density of 10,000 cells per well in 24 well culture plates (Falcon) for cell proliferation experiments and 50,000 cells per well for real-time PCR experiments with or without EMD as previously described. Three independent experiments were performed each with 3 replicates for each condition. Data were analyzed for statistical significance using 2-way analysis of variance (ANOVA) with Bonferroni test (*, p<0.05).

## Results

### Effect of EMD on Various Osteoblast-derived Cell Lines

**Figure 1:**
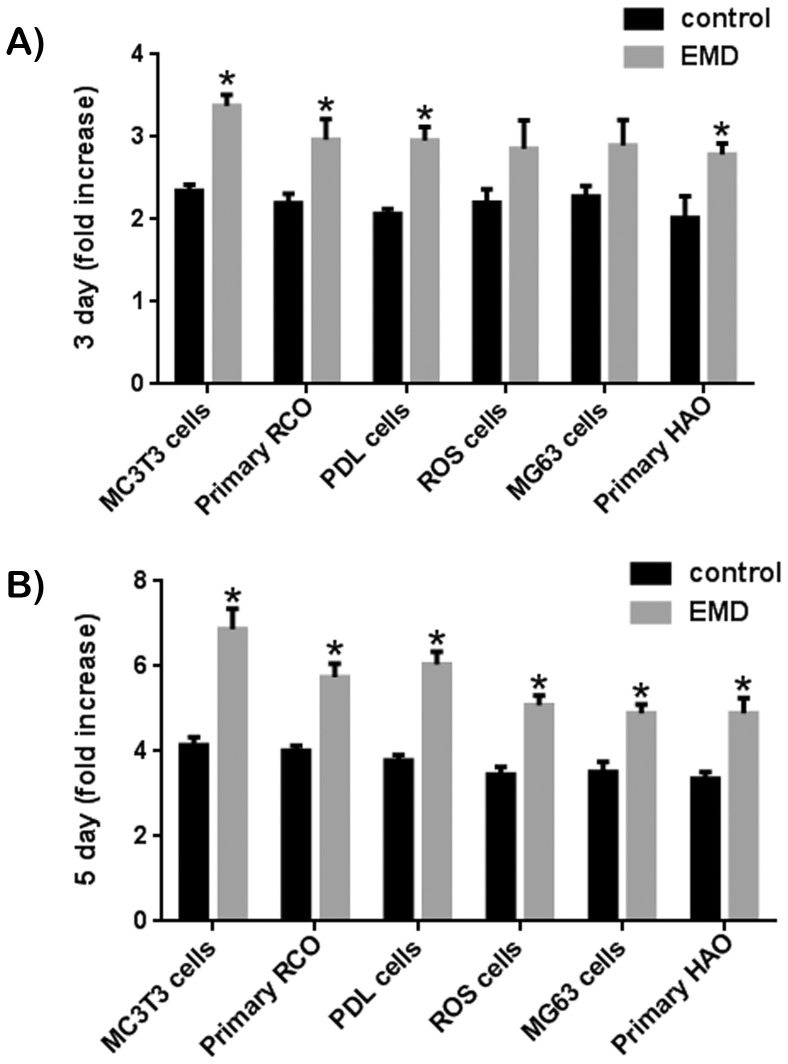
EMD significantly increases cell proliferation at (A) 3 and (B) 5 days post seeding for cell types including MC3T3-E1 pre-osteoblasts, primary rat calvarial osteoblasts (RCOs), primary human PDL cells, ROS osteoblast cell line, MG63 osteoblast cell line, and primary human alveolar osteoblasts (HAO).

**Figure 2:**
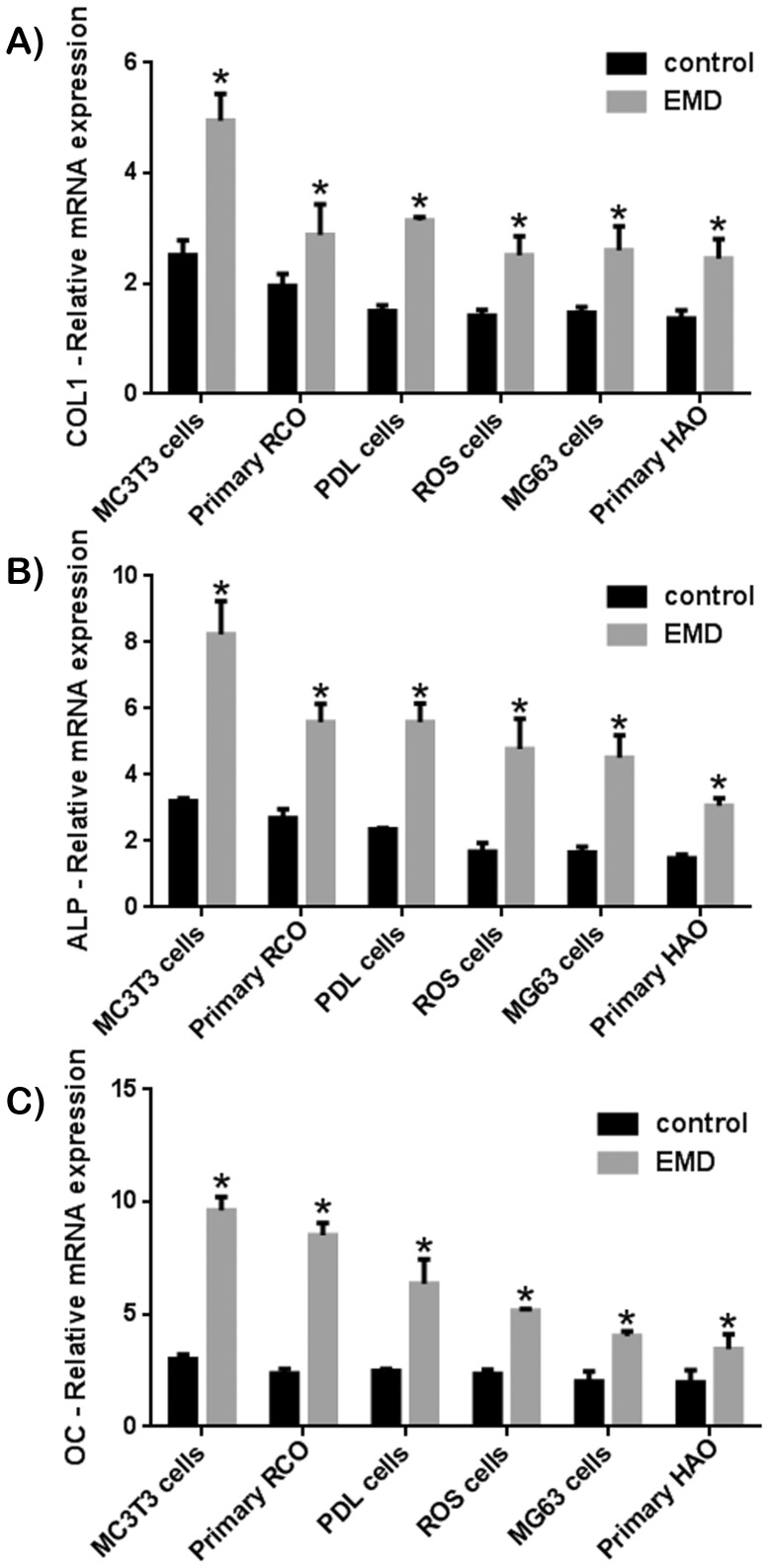
Effects of EMD on mRNA levels of osteoblast differentiation markers including (A) collagen1 (*COL1*), (B) alkaline phosphatise (*ALP*) and (C) osteocalcin (*OC*) as assessed by real-time PCR at 14 days post seeding. EMD significantly increased *COL1*, *ALP* and *OC* for all cell types with large variability. EMD increased mRNA levels of osteoblast markers more prominently in cell types early in their differentiation process (MC3T3 pre-osteoblasts and PDL cells) when compared to mature osteoblasts (ROS and MG63 osteoblast cell lines, HAO).

**Figure 3:**
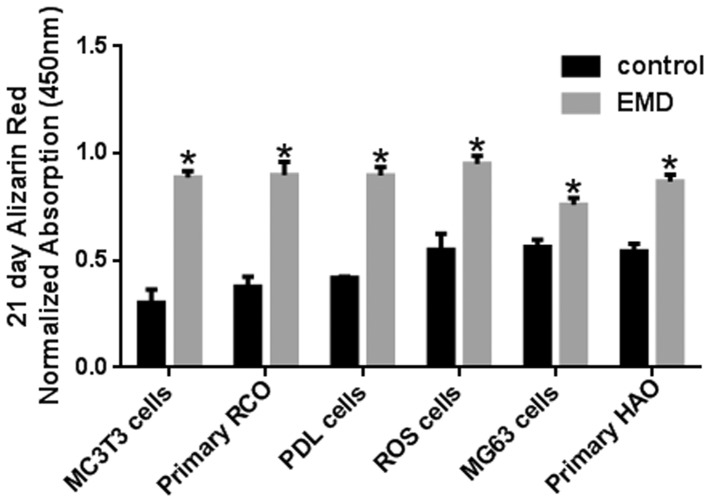
Alizarin red staining (ARS) of various cell types receiving treatment with EMD. A significant increase in ARS staining was observed in all cell types treated with EMD.

The impact of EMD was first tested on the proliferative capacity of various cell types (Fig. 1). At 3 days post seeding (Fig. 1A), EMD showed a significant effect on 4 of the 6 cell types. The 2 osteoblast derived cell-lines MG63 and ROS cells demonstrated a non-significant increase in cell proliferation. By 5 days post seeding (Fig. 1B), EMD significantly upregulated all cell types irrespective of their species origin and differentiation state. Little differences were observed with respect to their proliferative potential. Following cell proliferation, each cell type was exposed to EMD and evaluated for osteoblast differentiation by assessing mRNA levels of genes encoding *COL1*, *ALP* and *OC* (Fig. 2). EMD significantly increased *COL1* expression, however a larger increase was observed in MC3T3-E1 pre osteoblast cell-line compared to the other cell types (Fig. 2A). Similarly, EMD significantly increased mRNA levels of *ALP* in all cell types. However, here a slight increase in *ALP* mRNA levels was observed for cells of an earlier lineage including MC3T3-E1 cells, primary RCOs and human PDL cells when compared to mature osteoblasts (Fig. 2B). This effect was further supported by *OC* mRNA data where almost a 4 fold increase was observed for MC3T3-E1 cells treated with EMD compared to its control whereas less than a 2 fold increase was observed in mature osteoblast cell lines including ROS and MG63 cells and primary HAO (Fig. 2C). Figure 3 demonstrates alizarin red (AR) staining for all groups treated with EMD following 21 days incubation. Control cells earlier in their differentiation stages (MC3T3-E1 cell-line and RCO) showed lower levels of AR staining when compared to osteoblasts of a more differentiated phenotype (Fig. 3). Although the effects of EMD were more pronounced in cells earlier in their differentiation stages (MC3T3-E1 cell-line and RCO), the mineralization as demonstrated via AR staining seamed to reach a plateau for all cell types (Fig. 3).

### Effect of PDL Cell Passaging on Responsiveness to EMD Treatment

**Figure 4:**
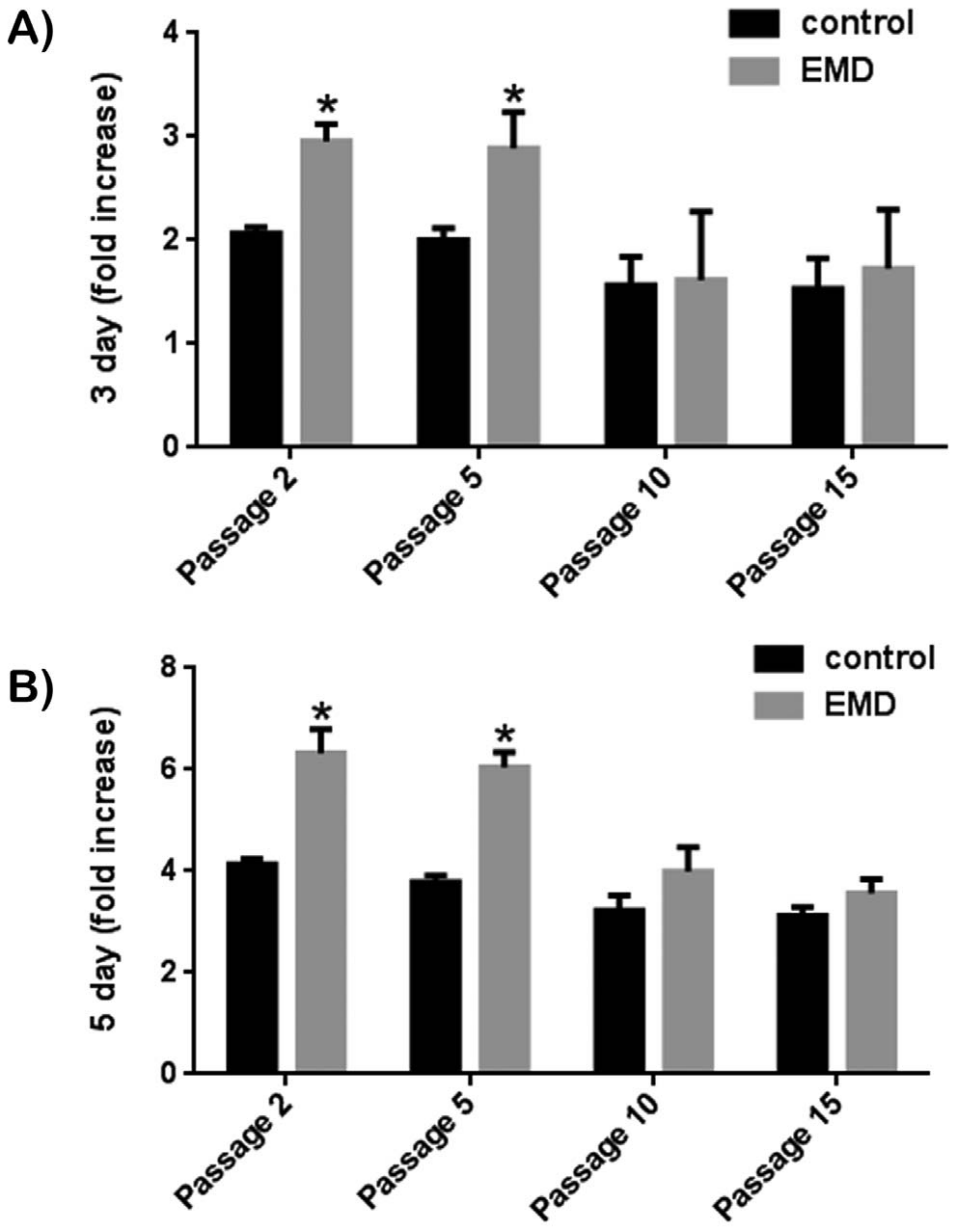
Effect of EMD on primary human PDL cells passaged 2, 5, 10 and 15 times on cell proliferation at (A) 3 and (B) 5 days post seeding. EMD significantly increased cell proliferation of cells passaged up to passage 5. For cell passage 10 and 15, a significant increase in cell proliferation was no longer observed at either 3 or 5 days post seeding.

**Figure 5:**
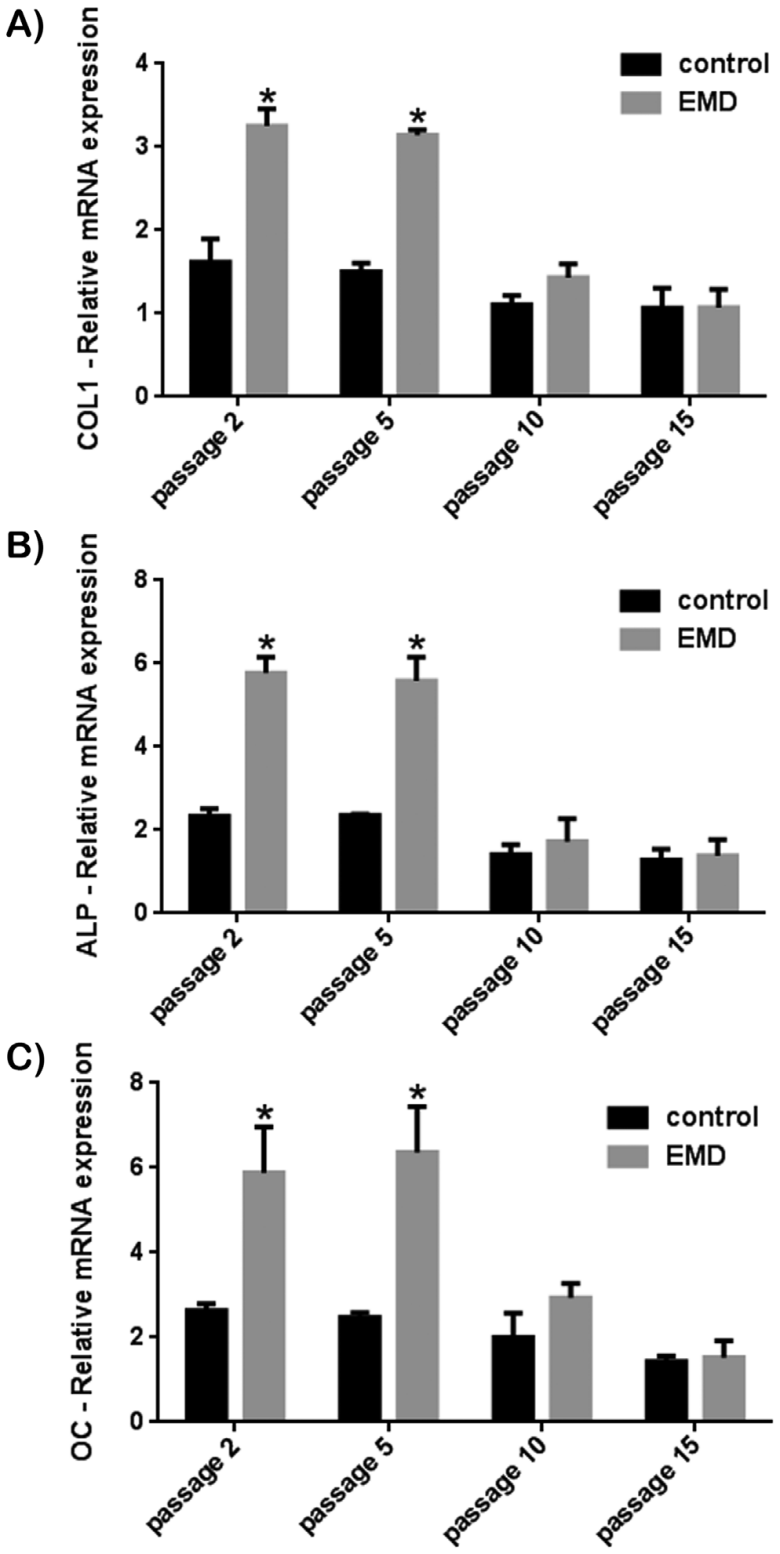
Effect of EMD on primary human PDL cell mRNA expression of genes encoding (A) *COL1*, (B) *ALP* and (C) *OC*. EMD significantly increased all osteoblast differentiation markers at passages 2 and 5, however failed to increase markers *COL1*, *ALP* or *OC* at passages 10 and 15 days.

In order to determine the effects of over-passaging primary cells in response to treatment with EMD, primary human PDL cells were exposed to EMD treatment following cell passages 2, 5, 10 and 15 (Fig. 4, 5). PDL cells passaged 2 and 5 times revealed a significant increase in cell proliferation at 3 and 5 days post seeding in response to EMD (Fig. 4A, B). Cells that were passaged 10 and 15 times were no longer able to respond to treatment with EMD. A similar effect was also observed on mRNA levels of genes encoding *COL1*, *ALP* and *OC* (Fig. 5). EMD significantly upregulated all osteoblast differentiation markers for cells passaged 2 and 5 times, however when cells were passaged 10 or more times, PDL cells were no longer able to respond to treatment with EMD (Fig. 5). Furthermore, in control samples not coated with EMD, expression of ALP and OC had both been decreased by 2 fold when compared to control samples at passages 2 demonstrating not only a negative response to treatment with EMD but also a loss of function and responsiveness to regular cell culture conditions.

### Effect of EMD on MC3T3-E1 Pre-osteoblasts at Various Differentiation Stages

**Figure 6:**
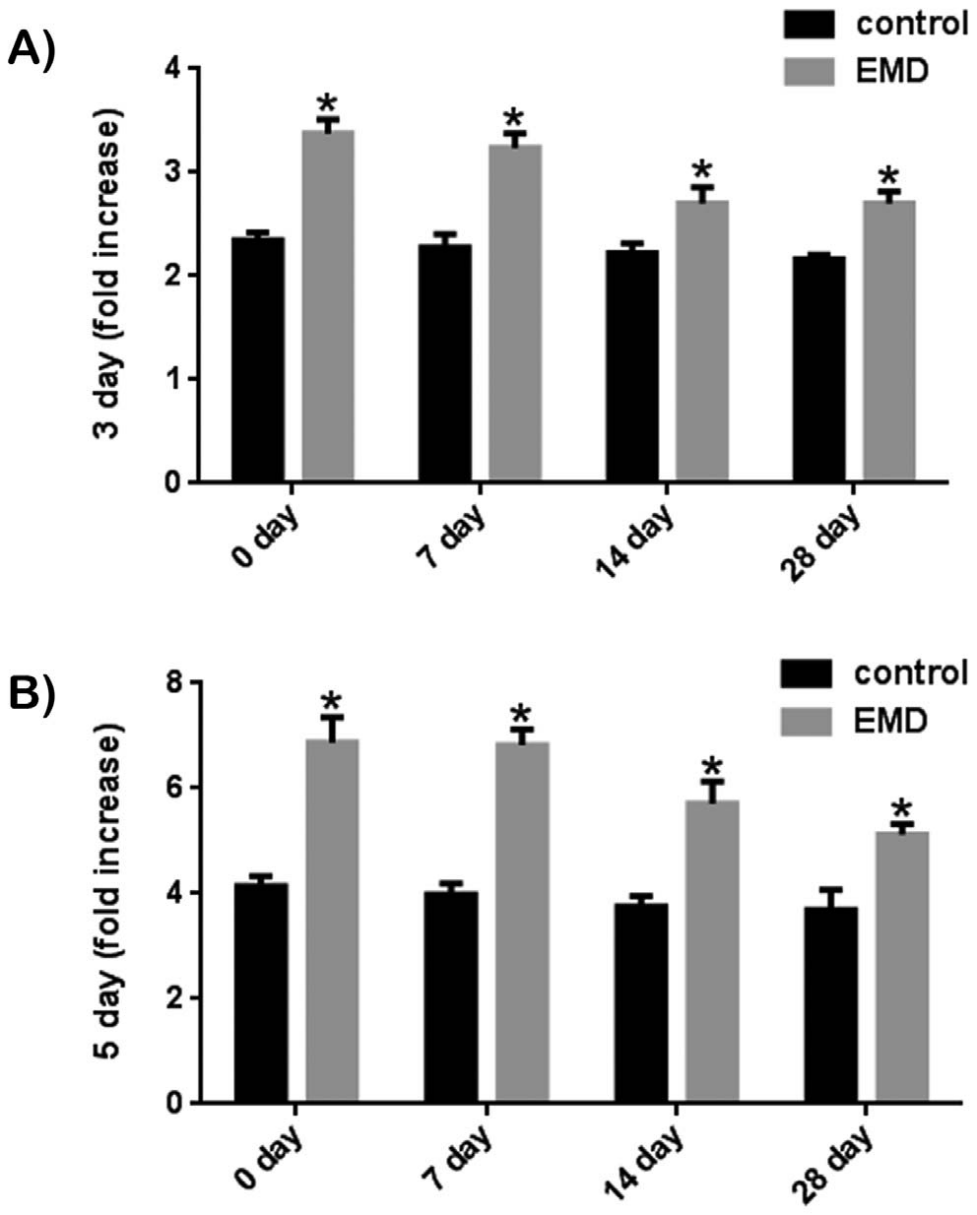
MC3T3-E1 pre-osteoblasts were left in T-75 flasks for 0, 7, 14 or 28 days to induce osteoblast differentiation via cell-cell contacts prior to cell seeding. EMD was able to increase cell proliferation significantly at both (A) 3 and (B) 5 days post seeding.

**Figure 7:**
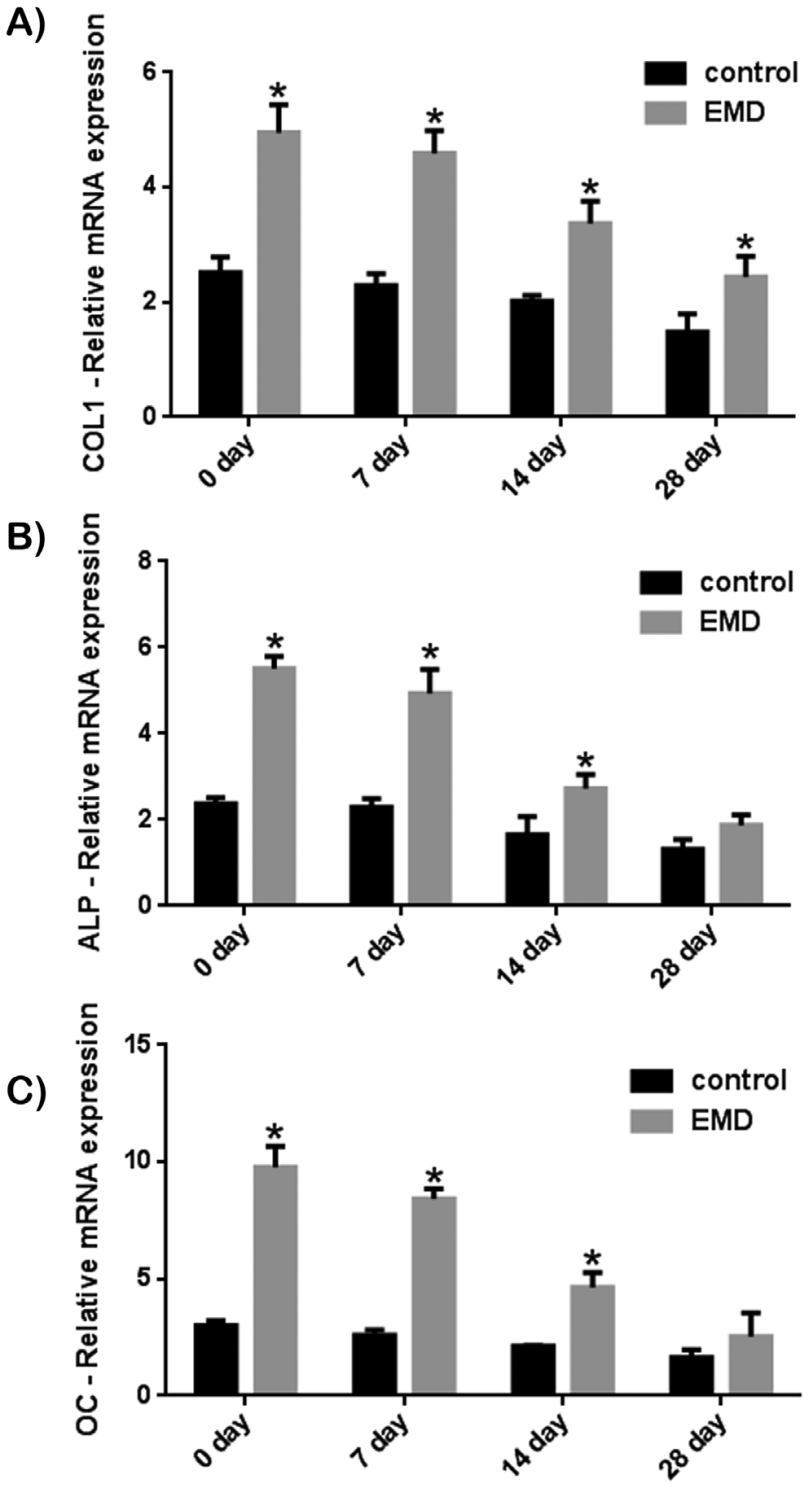
mRNA levels of (A) *COL1*, (B) *ALP* and (C) *OC* of MC3T3-E1 pre-osteoblasts that were left in T-75 flasks for 0, 7, 14 or 28 days to induce osteoblast differentiation via cell-cell contacts prior to cell seeding. EMD significantly increased cell differentiation for all osteoblast markers up to 14 days. At 28 days, only *COL1* was significantly up-regulated. The effects of EMD on MC3T3 pre-osteoblasts decreased as cells were left in flasks from 0 to 28 days.

This experiment sought to determine the effect of EMD on a pre-osteoblast cell line and to monitor its response as cells were gradually differentiated down an osteoblast phenotype (Fig. 6 and 7). MC3T3-E1 mice pre-osteoblast cell line were gradually differentiated towards the osteoblast phenotype via spontaneous differentiation induced by cell-cell contacts once confluency was reached under standard in vitro tissue culture conditions for a period ranging from 0 to 28 days (Figure S2). The effects of EMD on the proliferation potential of MC3T3 cells was not significantly altered for cells under standard tissue culture conditions from 0 to 28 days at either 3 or 5 days post seeding (Fig. 6). EMD was able to significantly increase cell proliferation at all time points irrespective of the number of days cells remained in confluency prior to EMD treatment. The effect of EMD on MC3T3 cells was then tested for cell differentiation by assessing mRNA levels of *COL1*, *ALP* and *OC* (Fig. 7). In this experiment, a time dependant effect was observed for MC3T3 cells having been left in confluency from 0 to 28 days. A significant decrease in the cells ability to produce *ALP* and *OC* was significantly reduced following 28 days in standard culture medium without passage prior to treatment with EMD (Fig. 7).

## Discussion

EMD is a widely used biologic agent capable of enhancing periodontal wound healing/regeneration [4]. In a systematic review on the effects of enamel matrix proteins, it was demonstrated that EMD has a significant influence on cell adhesion, cell proliferation and cell differentiation of many cell types by mediating cell attachment, spreading, proliferation and survival as well as expression of transcription factors, growth factors, cytokines, extracellular matrix constituents and other molecules involved in the regulation of bone remodelling [46].

One of the aims of the present study was to investigate the effect of cell maturation on the response to treatment with EMD. To achieve this goal, 6 osteoblast cell types, progenitor cells and cell-lines were utilized ranging from MC3T3-E1 mice pre-osteoblast progenitor cells, to differentiated osteoblast cell lines including ROS and MG63 cells. In the present study, we have demonstrated that EMD was able to significantly upregulate proliferation of all cell types at 5 days post seeding (Fig. 1). Although little difference between cell types was observed in cell proliferation rates following treatment with EMD, the effects of EMD on osteoblast differentiation was significantly altered. mRNA levels of *OC*, a late marker for osteoblast differentiation, was significantly increased almost 4 fold in MC3T3 pre-osteoblasts, whereas the effects of EMD on mRNA expression of *OC* on fully matured osteoblasts increased less then 2 fold in ROS, MG63 and HAO (Fig. 2C).

The finding that EMD increased MC3T3-E1 cells is consistent with findings from other studies in the literature [40], [47]–[50]. To date, a total of 5 studies have tested the effect of EMD on either cell proliferation or differentiation of MC3T3-E1 pre-osteoblasts [40], [47]–[50]. In each of these studies, EMD significantly increased cell proliferation and markers for osteoblast differentiation [40], [47]–[50]. Weishaupt et al. demonstrated that the mRNA expression of bone sialoprotein (*BSP*), also a late marker for osteoblast differentiation, was upregulated 13.9 times in comparison to control samples after 16 days of culture when exposed to EMD [50]. Although *BSP* was not tested in this study, the result that EMD was most able to promote osteoblast differentiation of MC3T3 cells was also shown. Interestingly, in the present study it was observed that EMD also had a pronounced effect on PDL cell differentiation down the osteoblast lineage. In a number of previous in vitro experiments from our laboratory determining the effects of EMD in combination with various bone grafting materials [51]–[54], it was consistently found that human PDL cell responded more preferably to EMD when compared to osteoblasts treated with EMD in the same in vitro conditions [51], [53]. In light of these results and with the combination of the current experiments, an accumulation of evidence provides more support to the fact that EMPs target cells earlier in their differentiation process.

The second aim of this study was to determine the effect of EMD on primary human PDL cells having been passaged 2, 5, 10 or 15 times. To the best of our knowledge, to date no study has compared the influence of cell passaging of primary human cells in response to treatment with EMD. In the present study it was observed that cells passaged 2 and 5 times responded favourably to treatment with EMD but PDL cells passaged 10 and 15 times were no longer able to proliferate or differentiate in response to EMD (Fig. 4, 5). The results from the present study thus showed that primary cells having been passaged more then 5 times may lose their inherent properties to differentiate and proliferate in response to EMD and potentially other growth factors. While a large number of studies have tested the response of EMD to primary human cells, a number of them have failed to report up to which passage number these cells are utilized. The results from the present study suggest that this information may have major implications on in vitro cell response.

In the third set of studies, MC3T3-E1 cells were utilized because of their undifferentiated phenotype as well as their ability to respond to EMD both in previous studies and also in this study. It has previously been reported that cells having undergone cell-cell contacts are able to gradually differentiate down the osteoblastic lineage [44], [45]. Because in vitro cell maintenance conditions are rarely reported in the literature, this method of induction down the osteoblast lineage was chosen as opposed to inducing osteoblast differentiation via differentiation factors including ascorbic acid, beta-glycerophosphate and/or dexamethasone [55], [56] to demonstrate the ability of cells to lose their potential to respond to treatment from EMD. In this study, a time-dependant reduction in osteoblast differentiation was observed when cells were left unpassaged from 7 to 28 days following treatment with EMD. Interestingly, we have previously reported that EMD enhances osteoblast differentiation via cell-cell contacts by upregulating osteoblast gap junctional proteins, connexin 43 (*cx43*) [32]. Gap junctions are aqueous transmembrane channels that connect the cytoplasm of two adjacent cells and allow the diffusion of small molecules with a molecular mass of less than 1 kDa such as small metabolites, ions, and intracellular signaling molecules (calcium, cAMP, and inositol triphosphate) to pass through [57]. *Cx43* molecules are absolutely essential for osteoblast differentiation and function [58]–[61] and EMD targets their upregulation [32]. The results from the present study may also indicate that *cx43* may be implicated in the down-regulation of osteoblast differentiation in response to EMD following cell maturation. However, this hypothesis requires further experimental investigations.

### Conclusions

The results from the present study suggest that EMD has a more pronounced effect on cells earlier in their differentiation process. The effects on mRNA levels of osteocalcin were most prominent on MC3T3-E1 pre-osteoblasts, primary RCOs and PDL cells and gradually decreased to a more mature osteoblast phenotype. Furthermore, primary human PDL cells passaged 10 or more times were no longer able to respond to treatment with EMD and the same observation was observed for MC3T3 pre-osteoblasts that had undergone osteoblast differentiation via cell-cell contacts. The results from the present study may also provide support for the clinical and histological observations that EMD seems to favour regeneration of periodontal tissues including the periodontal ligament which contains a high proportion of undifferentiated progenitor cells when compared to pure alveolar bone defects.

## Supporting Information

Figure S1 **MC3T3 cells, primary RCO, PDL cells, ROS cells, MG63 cells and primary HAO were analyzed for mRNA expression of osteoblast differentiation markers A) COL1, B) ALP, and C) OC at baseline prior to all experiments to confirm their maturation state.** A) Although no significant difference was observed for COL1 expression, cell-lines ROS and MG63 as well as primary HAO demonstrated higher levels at baseline for genes encoding COL1 as assessed by real-time PCR. B) A significant increase in genes encoding ALP and OC were observed in cell-lines ROS and MG63 as well as HAO at baseline when compared to MC3T3 pre-osteoblasts, primary RCO and human primary PDL cells. The results from the present experiment demonstrates higher expression of osteoblast-related differentiation markers in human primary osteoblasts and osteosarcoma-derived cell lines when compared to pre-cursor cells derived from a mesenchymal origin (MC3T3 cells), neonatal rat calvaria and PDL cells. (*, p<0.05, results from 3 independent experiments).
(TIF)

Figure S2 **MC3T3 cells were left in T-75 flasks for 0, 7, 14 and 28 days to gradually differentiate cells towards the osteoblast lineage via spontaneous differentiation induced by cell-cell contacts once confluency was reached under standard in vitro conditions.** At time points 0, 7, 14 and 28 days, cells were analyzed for mRNA expression of osteoblast differentiation markers A) COL1, B) ALP, and C) OC prior to application with EMD to confirm the differentiation of pre-osteoblasts down the osteoblast lineage. A non-significant increase in mRNA levels of COL1 and ALP was observed from 0 to 28 days demonstrating the gradual increased expression of osteoblast-related markers in the absence of osteoblast differentiation media. A significant increase in OC, a late marker for osteoblast differentiation, was observed 14 days post-confluency, and a 3.5 fold significant increase was observed. (*, p<0.05, **, p<0.05 above all other values, results from 3 independent experiments).
(TIF)
