# Supplementary figures and images for: Influence of Enamel Matrix Derivative on Cells at Different Maturation Stages of Differentiation
Source: PLoS One. 2013 Aug 12;8(8):e71008. doi: 10.1371/journal.pone.0071008 (PMC3741386; doi:10.1371/journal.pone.0071008)

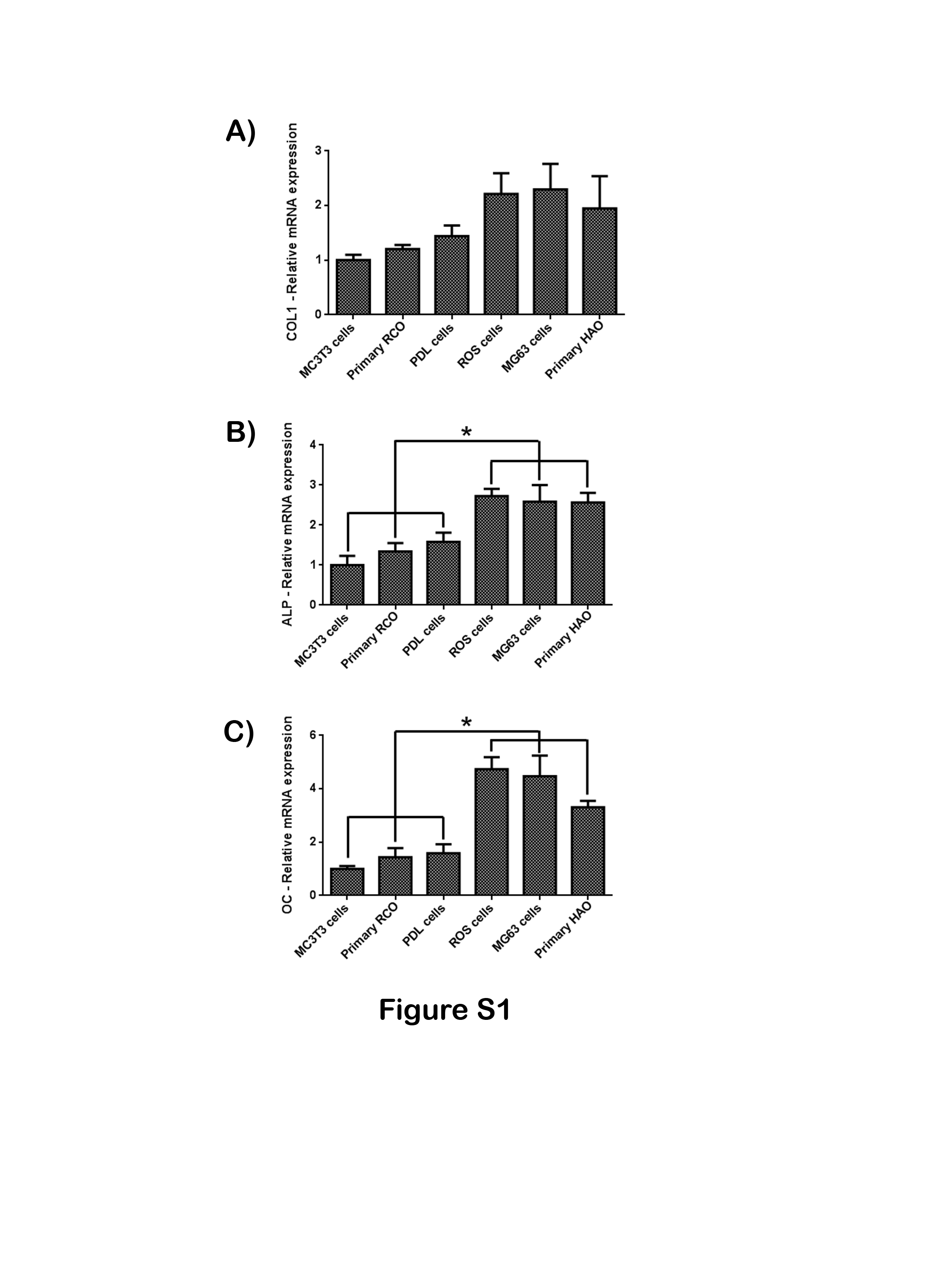

Supplement: Figure S1 — MC3T3 cells, primary RCO, PDL cells, ROS cells, MG63 cells and primary HAO were analyzed for mRNA expression of osteoblast differentiation markers A) COL1, B) ALP, and C) OC at baseline prior to all experiments to confirm their maturation state. A) Although no significant difference was observed for COL1 expression, cell-lines ROS and MG63 as well as primary HAO demonstrated higher levels at baseline for genes encoding COL1 as assessed by real-time PCR. B) A significant increase in genes encoding ALP and OC were observed in cell-lines ROS and MG63 as well as HAO at baseline when compared to MC3T3 pre-osteoblasts, primary RCO and human primary PDL cells. The results from the present experiment demonstrates higher expression of osteoblast-related differentiation markers in human primary osteoblasts and osteosarcoma-derived cell lines when compared to pre-cursor cells derived from a mesenchymal origin (MC3T3 cells), neonatal rat calvaria and PDL cells. (*, p<0.05, results from 3 independent experiments). (TIF) [file pone.0071008.s001.tif]

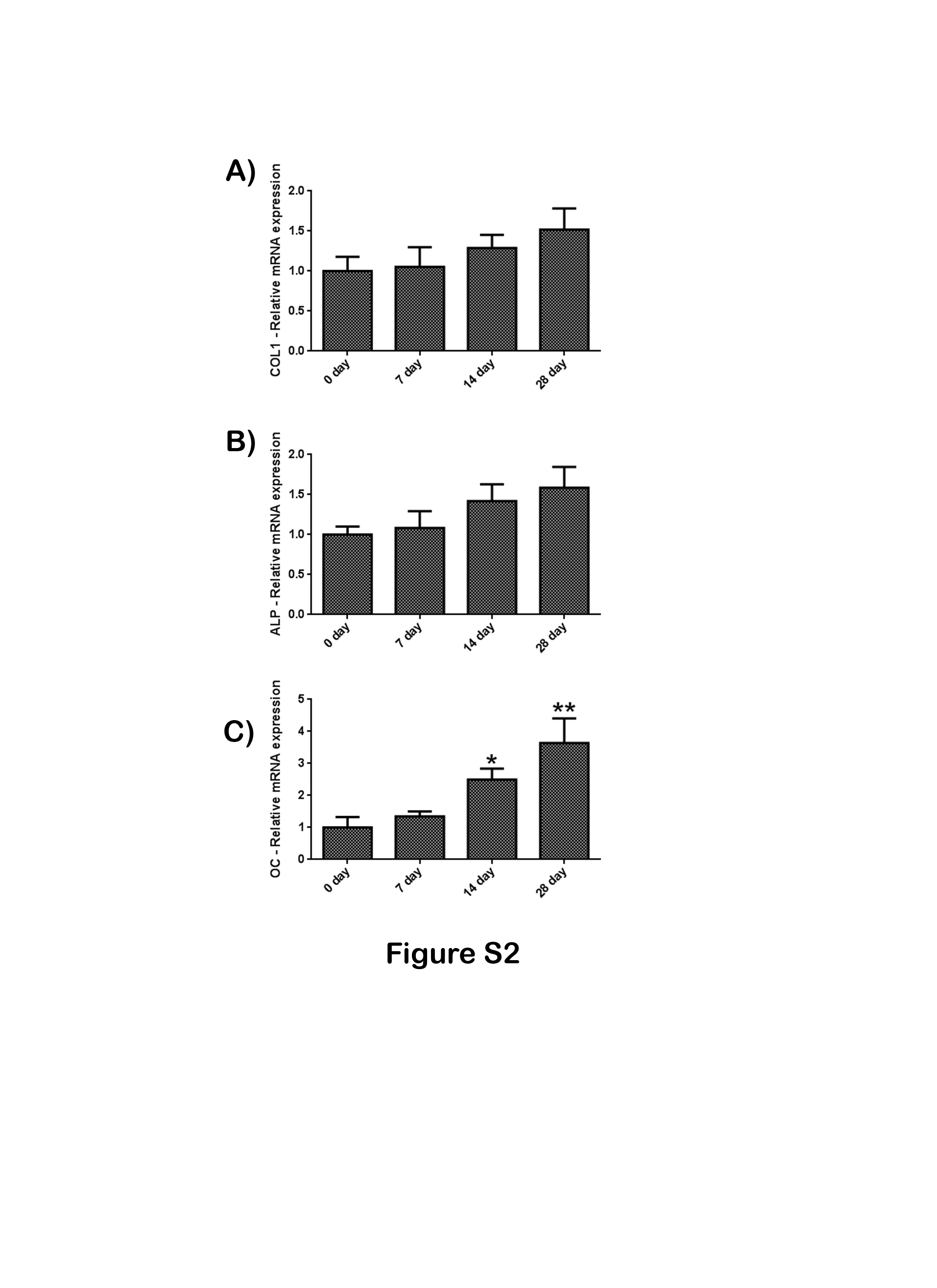

Supplement: Figure S2 — MC3T3 cells were left in T-75 flasks for 0, 7, 14 and 28 days to gradually differentiate cells towards the osteoblast lineage via spontaneous differentiation induced by cell-cell contacts once confluency was reached under standard in vitro conditions. At time points 0, 7, 14 and 28 days, cells were analyzed for mRNA expression of osteoblast differentiation markers A) COL1, B) ALP, and C) OC prior to application with EMD to confirm the differentiation of pre-osteoblasts down the osteoblast lineage. A non-significant increase in mRNA levels of COL1 and ALP was observed from 0 to 28 days demonstrating the gradual increased expression of osteoblast-related markers in the absence of osteoblast differentiation media. A significant increase in OC, a late marker for osteoblast differentiation, was observed 14 days post-confluency, and a 3.5 fold significant increase was observed. (*, p<0.05, **, p<0.05 above all other values, results from 3 independent experiments). (TIF) [file pone.0071008.s002.tif]
